# Supplementary material for: Permutation-based identification of important biomarkers for complex diseases via machine learning models
Source: Nat Commun. 2021 May 21;12:3008. doi: 10.1038/s41467-021-22756-2 (PMC8140109; doi:10.1038/s41467-021-22756-2)
Supplement: Supplementary file 1 — Supplementary Information [file 41467_2021_22756_MOESM1_ESM.pdf]

Supplementary Information: Permutation-based Identification of Important Biomarkers for Complex Diseases via Machine Learning Models

Xinlei Mi[1], Baiming Zou[2], Fei Zou[2], & Jianhua Hu[3*]

March 17, 2021

1 Department of Preventive Medicine, Northwestern University

2 Department of Biostatistics, University of North Carolina at Chapel Hill

3 Department of Biostatistics, Columbia University

* Corresponding author: jh3992@cumc.columbia.edu

ρ	Variable	$N = 1000, p = 100$									$N = 5000, p = 100$								
		PermFIT DNN	HRT DNN	PermFIT RF	Vanilla RF	PermFIT SVM	SHAP* DNN	LIME* DNN	SNGM* DNN	RFE* SVM	PermFIT DNN	HRT DNN	PermFIT RF	Vanilla RF	PermFIT SVM	SHAP* DNN	LIME* DNN	SNGM* DNN	RFE* SVM
0	X_1	100	100	100	100	100	100	100	100	100	100	100	100	100	100	100	100	100	100
	X_{p_0+1}	100	100	100	100	84	100	16	35	9	100	100	100	100	100	100	14	45	11
	X_{2p_0+1}	100	100	100	100	100	100	91	100	100	100	100	100	100	100	100	68	100	100
	X_{3p_0+1}	100	100	53	80	71	98	16	38	8	100	100	100	100	100	100	10	38	6
	X_{4p_0+1}	100	100	52	75	73	97	9	40	6	100	100	100	100	100	100	11	40	6
	S_0	5.7	7.0	5.0	13.9	5.0	5.1	10.4	7.2	8.5	4.5	4.9	3.7	13.1	4.7	5.8	11.6	6.9	8.4
	S_1	5.0	7.4	5.1	13.8	5.3	5.5	6.0	7.3	7.9	4.8	5.0	4.3	13.3	5.2	4.8	5.5	7.3	8.0
0.2	X_1	100	100	100	100	100	100	100	100	100	100	100	100	100	100	100	100	100	100
	X_{p_0+1}	100	100	100	100	84	100	13	44	1	100	100	100	100	100	100	11	42	0
	X_{2p_0+1}	100.	100	100	100	100	100	89	100	100	100	100	100	100	100	100	70	100	100
	X_{3p_0+1}	100	100	60	80	81	96	13	41	2	100	100	100	100	100	100	9	41	0
	X_{4p_0+1}	100	100	54	80	77	99	10	50	2	100	100	100	100	100	100	10	36	0
	S_0	6.1	6.5	7.8	28.8	6.3	5.5	11.0	7.2	16.4	5.6	5.1	17.8	51.4	9.3	5.5	11.3	7.4	17.8
	S_1	5.5	7.6	5.0	14.5	4.7	5.2	5.6	6.8	1.2	4.6	5.4	5.7	16.1	4.7	5.0	5.8	7.0	0.0
0.5	X_1	100	100	100	100	100	100	100	100	100	100	100	100	100	100	100	100	100	100
	X_{p_0+1}	100	100	100	100	100	100	20	47	0	100	100	100	100	100	100	22	32	0
	X_{2p_0+1}	100	100	100	100	100	100	91	100	100	100	100	100	100	100	100	66	100	100
	X_{3p_0+1}	100	100	65	90	90	98	11	36	0	100	100	100	100	100	100	9	41	0
	X_{4p_0+1}	100	100	64	87	87	99	8	45	0	100	100	100	100	100	100	6	31	0
	S_0	10.4	7.4	28.4	69.1	14.0	6.5	11.1	7.8	17.8	7.9	5.6	79.6	89.3	31.1	5.8	12.5	7.7	17.8
	S_1	5.9	7.2	5.2	19.5	5.2	4.2	5.4	6.4	0.0	6.0	5.6	11.8	34.1	8.9	4.8	4.7	7.0	0.0
0.8	X_1	100	98	100	100	100	100	100	100	100	100	100	100	100	100	100	100	100	100
	X_{p_0+1}	100	100	100	100	98	100	8	40	0	100	100	100	100	100	100	17	39	0
	X_{2p_0+1}	100	100	100	100	100	100	82	100	98	100	100	100	100	100	100	56	100	100
	X_{3p_0+1}	100	91	91	98	88	92	15	33	0	100	100	100	100	100	100	7	30	0
	X_{4p_0+1}	100	92	90	99	84	89	10	39	0	100	100	100	100	100	100	5	38	0
	S_0	21.1	7.9	64.4	93.2	27.8	7.9	12.4	10.6	17.8	18.8	5.4	100.0	100.0	59.6	7.2	13.0	9.9	17.8
	S_1	8.1	7.7	6.5	36.1	7.6	3.2	4.5	4.2	0.0	6.1	4.8	32.3	83.7	28.0	3.5	4.6	5.0	0.0

Supplementary Table 1: Simulation Results on Binary Outcomes. Reported is the percentage of the important variables detected by each method (p-value cutoff of 0.05), out of 100 repetitions for each simulation scenario, for five true causal features: X_1, X_{p_0+1}, X_{2p_0+1}, X_{3p_0+1}, X_{4p_0+1}, and two null feature sets: S_0 and S_1. *: Note that SHAP-DNN, LIME-DNN, SNGM-DNN and RFE-SVM do not perform formal statistical testing, and features can only be ranked with no associated p-values. The reported results for each of these four methods are based on the top 10 selected features for a simple illustration. For PermFIT methods and Vanilla-RF, p-values are calculated from one-sided Z test.

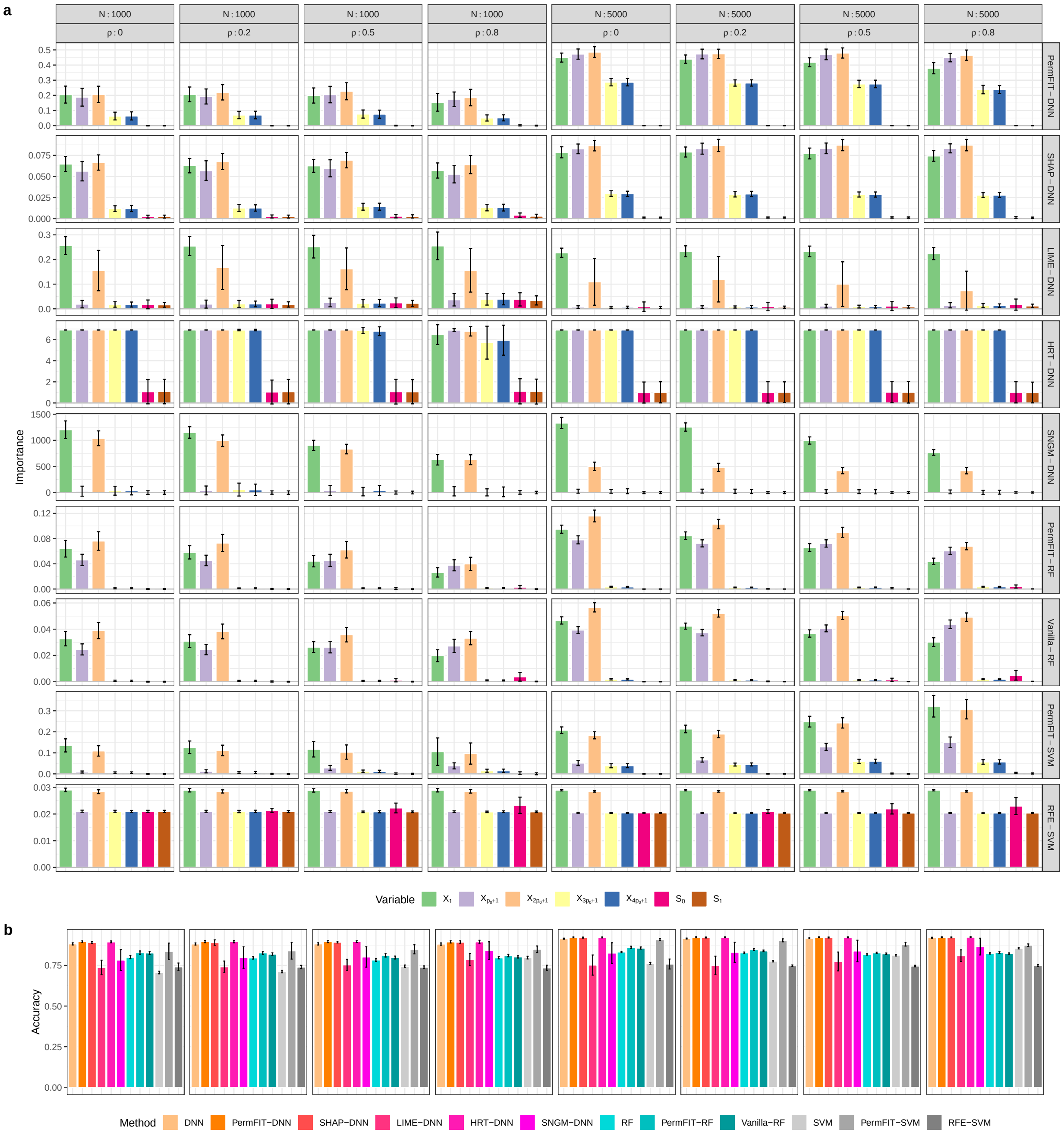

Supplementary Figure 1: Simulation Results on Binary Outcomes. a. Estimated feature importance for the five true causal features: X_1, X_{p_0+1}, X_{2p_0+1}, X_{3p_0+1}, X_{4p_0+1}, and two null feature sets: S_0, S_1. b. Prediction accuracy for methods in comparison. DNN, RF or SVM: specific modeling with all features; PermFIT-DNN, SHAP-DNN, LIME-DNN, HRT-DNN, SNGM-DNN, PermFIT-RF, Vanilla-RF, PermFIT-SVM or RFE-SVM: specific modeling after feature selection. Data are presented as mean values ± s.d. Simulations in each scenario are repeated for 100 times. Source data are provided as a Source Data file.

Method	DNN					RF		SVM	
	PermFIT	SHAP	LIME	HRT	SNGM	PermFIT	Vanilla	PermFIT	RFE
**TCGA kidney cancer data**									
Accuracy			0.704 (0.013)			0.694 (0.014)		0.690 (0.016)	
Accuracy*	0.751 (0.013)	0.731 (0.013)	0.650 (0.023)	0.750 (0.015)	0.724 (0.012)	0.732 (0.012)	0.713 (0.012)	0.744 (0.017)	0.709 (0.012)
AUC			0.753 (0.012)			0.753 (0.010)		0.752 (0.015)	
AUC*	0.820 (0.011)	0.800 (0.009)	0.694 (0.028)	0.816 (0.009)	0.793 (0.009)	0.808 (0.013)	0.781 (0.009)	0.815 (0.013)	0.781 (0.013)
**HITChip Atlas data**									
MSPE			0.990 (0.014)			0.995 (0.009)		1.019 (0.018)	
MSPE*	0.932 (0.018)	0.967 (0.015)	1.155 (0.030)	0.945 (0.016)	0.961 (0.014)	0.946 (0.009)	0.992 (0.009)	0.963 (0.025)	1.084 (0.012)
Correlation			0.492 (0.010)			0.490 (0.008)		0.478 (0.012)	
Correlation*	0.534 (0.013)	0.509 (0.011)	0.339 (0.033)	0.524 (0.011)	0.513 (0.010)	0.528 (0.007)	0.492 (0.008)	0.525 (0.017)	0.443 (0.008)

Supplementary Table 2: Model performance Improvement from Feature Selection: TCGA and Atlas. 5-fold cross-validated accuracy and AUC are evaluated for TCGA data, and 5-fold cross-validated correlation and MSPE are evaluated for Atlas data, randomly repeated for 100 time. Performance measurements are presented as mean values ± s.d. *: model performance with feature selection.

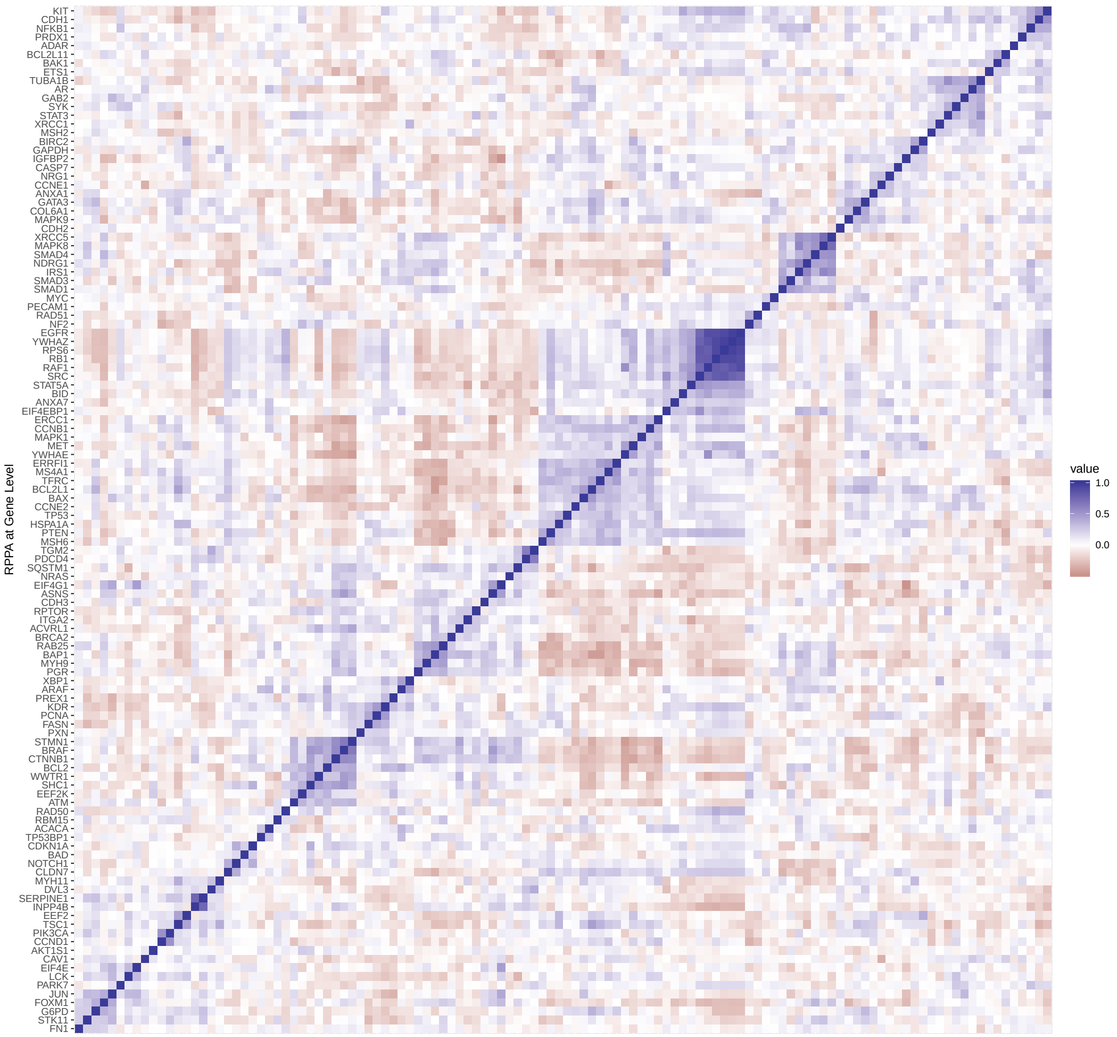

Supplementary Figure 2: Correlation map for TCGA RPPA features, ordered by hierarchical clustering. Source data are provided as a Source Data file.

Method	Runtime [mean (sd)]
PermFIT-DNN	40.4 (3.9)
SHAP-DNN	58.5 (7.7)
LIME-DNN	16.1 (2.2)
HRT-DNN	316.6 (59.3)
SNGM-DNN	40.1 (7.2)
PermFIT-RF	7.6 (0.7)
Vanilla-RF	0.8 (0.1)
PermFIT-SVM	9.5 (0.9)
RFE-SVM	128.0 (9.9)

Supplementary Table 3: Runtime (min) for each method in the simulation study with the continuous outcome at $N = 1000, p = 100, \rho = 0$.

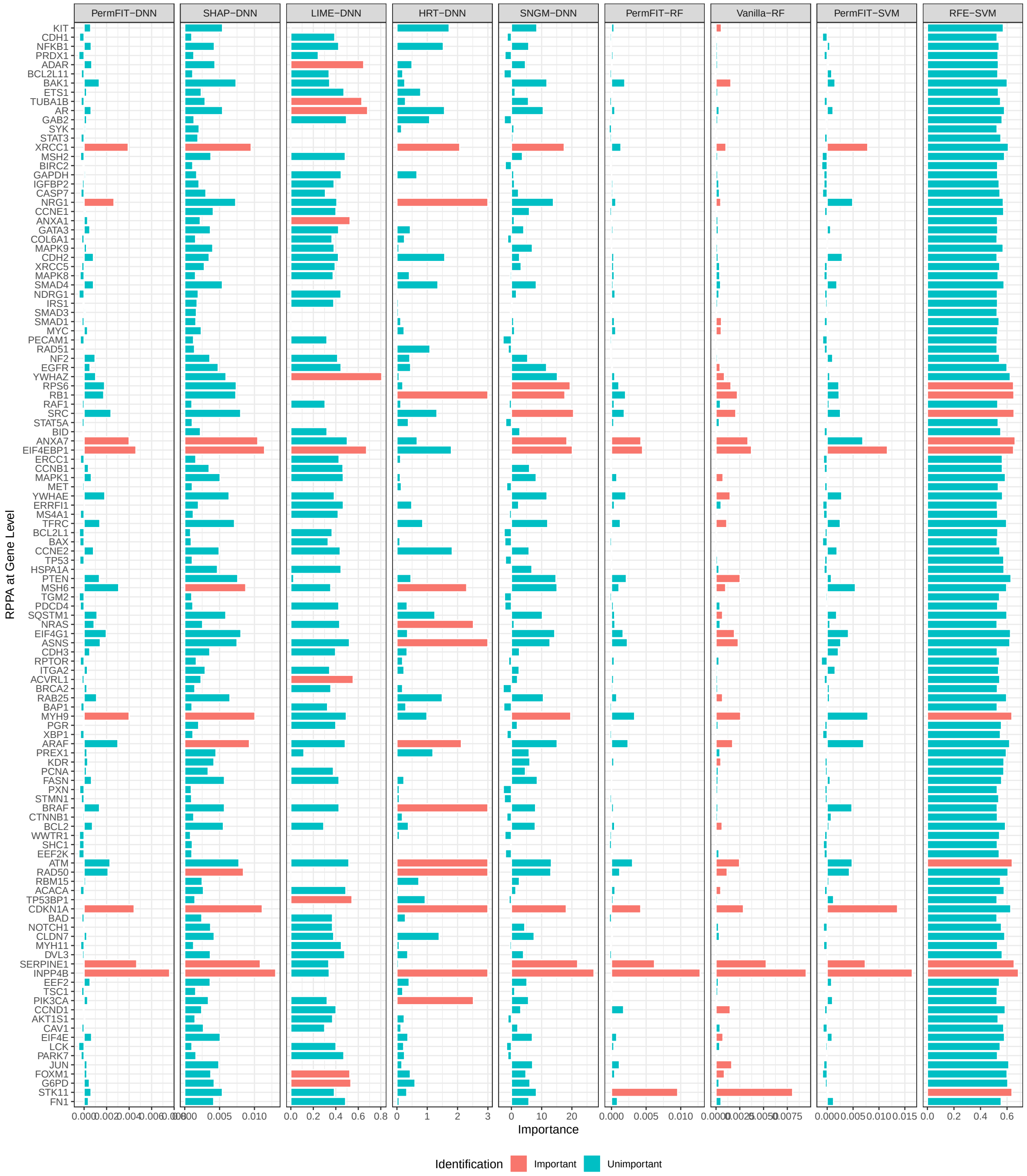

Supplementary Figure 3: Importance scores for TCGA kidney cancer data. Important features selected by each method is marked in red. PermFIT-DNN, HRT-DNN, PermFIT-RF, Vanilla-RF, PermFIT-SVM: p-values with FDR controlled at 0.1;SHAP-DNN, LIME-DNN, SNGM-DNN, RFE-SVM: Top 10 features. For HRT-DNN, $-\log_{10}$(p-value) is presented. Source data are provided as a Source Data file.

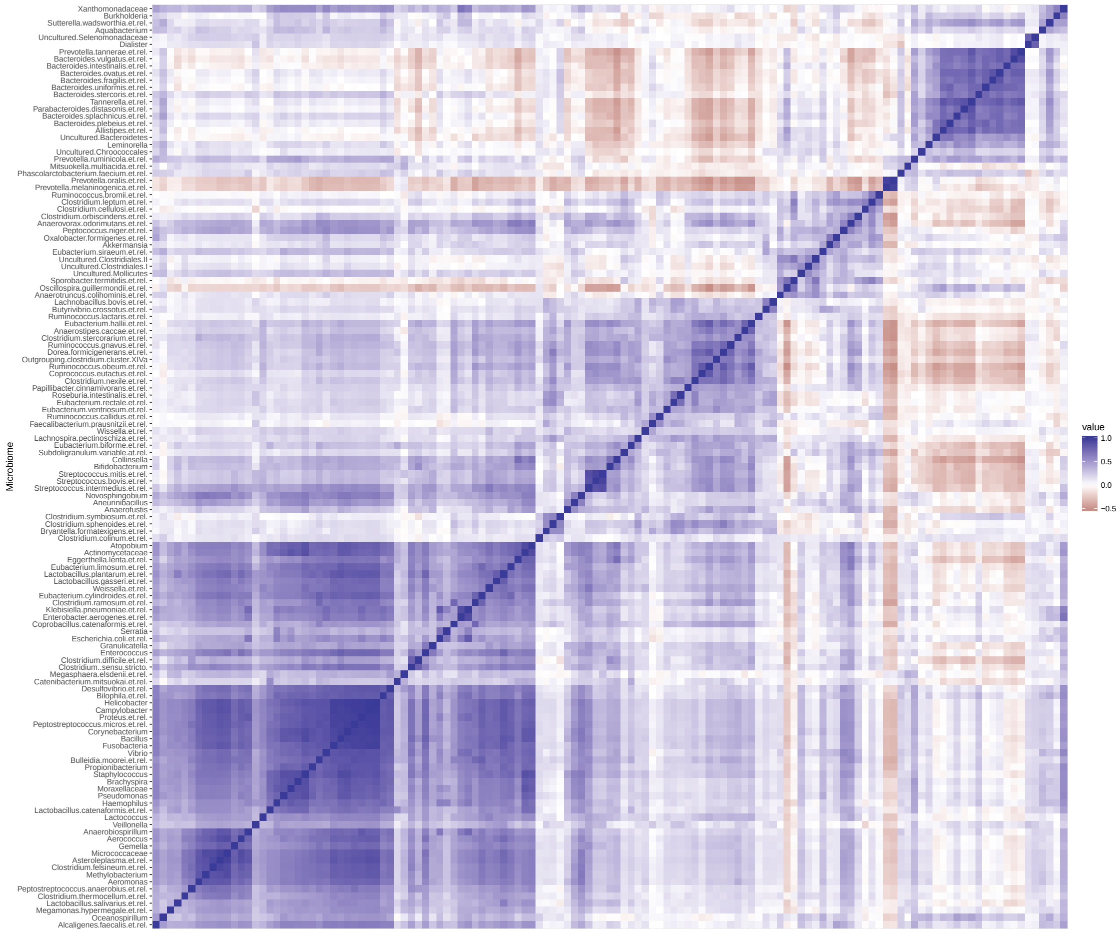

Supplementary Figure 4: Correlation map for HITChip Atlas microbiome features, ordered by hierarchical clustering. Source data are provided as a Source Data file.

Number of layers	MSPE		Correlation	
	DNN	PermFIT-DNN	DNN	PermFIT-DNN
1	1.580 (0.047)	1.281 (0.062)	0.830 (0.005)	0.862 (0.007)
2	1.506 (0.046)	1.254 (0.058)	0.839 (0.005)	0.865 (0.007)
3	1.496 (0.047)	1.270 (0.061)	0.840 (0.005)	0.864 (0.007)
4	1.503 (0.049)	1.279 (0.055)	0.839 (0.006)	0.863 (0.006)
5	1.517 (0.051)	1.278 (0.057)	0.838 (0.006)	0.864 (0.006)
6	1.532 (0.051)	1.278 (0.057)	0.836 (0.006)	0.864 (0.006)

Supplementary Table 4: Performance of DNN and PermFIT-DNN with different numbers of hidden layers in the DNN model, in the simulation study with the continuous outcome at $N = 1000, \rho = 0$.

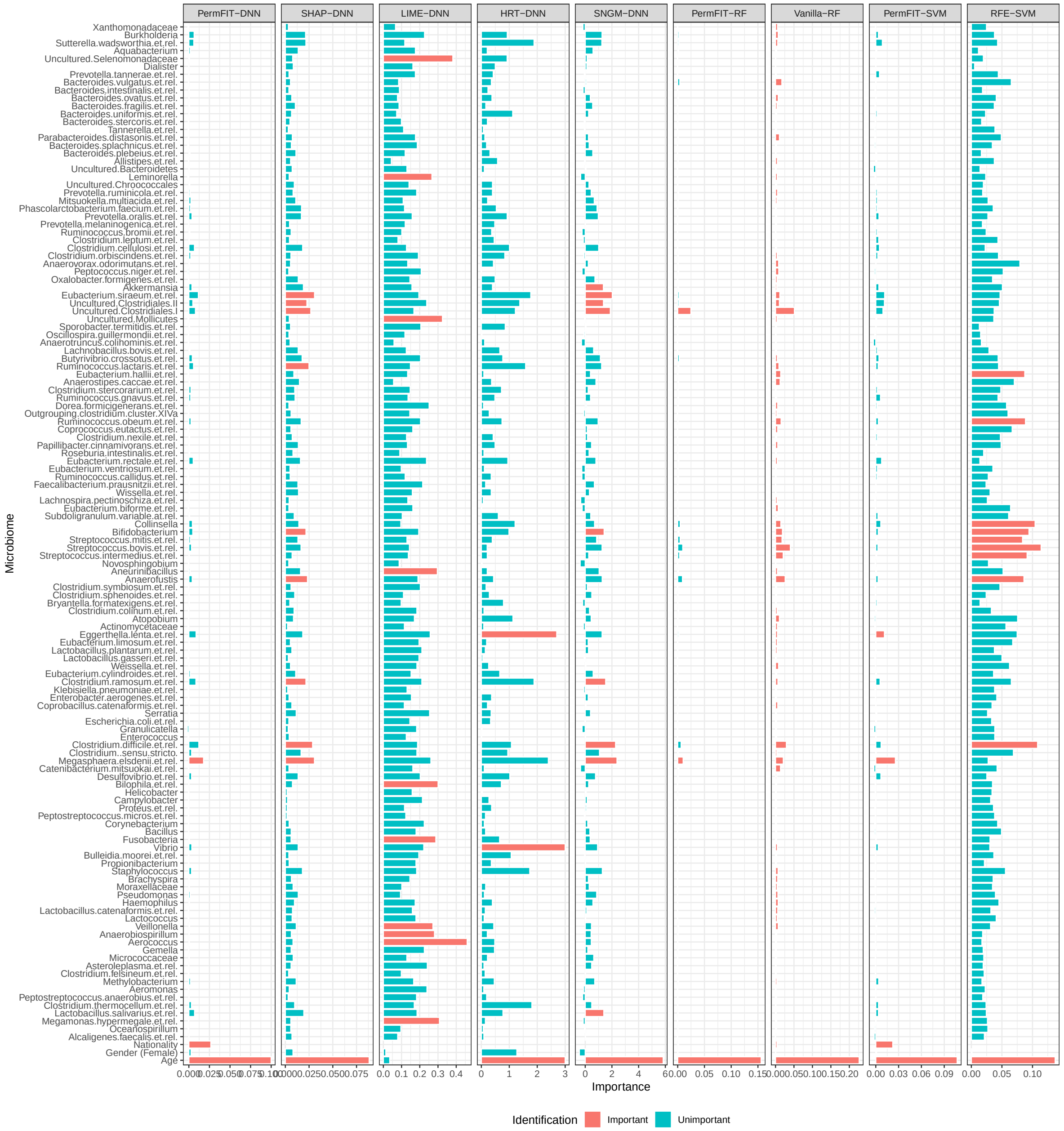

Supplementary Figure 5: Importance scores for HITChip Atlas data. Important features selected by each method is marked in red. PermFIT-DNN, HRT-DNN, PermFIT-RF, Vanilla-RF, PermFIT-SVM: p-values with FDR controlled at 0.1; SHAP-DNN, LIME-DNN, SNGM-DNN, RFE-SVM: Top 10 features. For HRT-DNN, $-\log_{10}$(p-value) is presented. Source data are provided as a Source Data file.
